# Therapeutic Potential of “Smart” Exosomes in Peripheral Nerve Regeneration

**DOI:** 10.26502/jbb.2642-91280082

**Published:** 2023-05-04

**Authors:** Rajiv Supra, Daniel R. Wilson, Devendra K Agrawal

**Affiliations:** 1 College of Osteopathic Medicine, Touro University, Henderson, Nevada, New York; 2 Department of Translational Research, College of Osteopathic Medicine of the Pacific, Pomona, California, USA

**Keywords:** Exosomes, Inflammation, Microvesicles, Nerve Regeneration, Nerve repair, Neurotherapeutic effect, Schwann cell

## Abstract

Peripheral nerve injury results in severe loss of motor and sensory function in the affected limb. The gold standard for peripheral nerve repair is autologous nerve grafts, but their inherent drawbacks limit their use. Satisfactory clinical data are yet to be obtained using tissue engineered nerve grafts with neurotrophic factors introduced in these grafts for nerve repair. Therefore, peripheral nerve regeneration still remains a challenge for clinicians. Exosomes are secreted nanovesicles from the extracellular membrane. They are critical for communication within the cell and play a crucial role in the pathologic process of the peripheral nervous system. Recent research supports the role of exosomes in exhibiting neurotherapeutic effects through axonal growth, Schwann cell activation, and regulating inflammation. Indeed, the use of “smart” exosomes by reprogramming or manipulating the secretome contents and functions are rising as a therapeutic option for treating peripheral nerve defects. This review provides an overview on the promising role of exosomes in the process of peripheral nerve regeneration.

## Introduction

Peripheral nerve injury result in lifelong disability that reduces the quality of life of more than one million people worldwide [1,2]. The peripheral nervous system is able to regenerate to a certain degree after peripheral nerve injury. The preferred method of peripheral nerve repair is through surgery using nerve grafts. Autologous nerve graft is the gold standard therapy for peripheral nerve injury [4,5]. The clinical use of autologous nerve grafts, however, has many drawbacks such as mismatch of length, neuroma formation, and availability of donor nerves [6]. Additionally, less than 50% of patients that undergone peripheral nerve graft surgery achieved sensory and motor recovery [7]. Natural synthetics and biomaterials have also been developed as potential substitutes for autologous nerve grafting, however, these substitutes fail to achieve satisfactory clinical results [8]. Although major advances have been made in the field of nerve regeneration, peripheral nerve repair still remains a challenge and exploring novel factors becomes critical to further improve the therapeutic effects of peripheral nerve injury. Exosomes are nano-sized vesicles that are secreted into the extracellular environment by neurons, osteocytes, and mesenchymal stem cells (MSCs) [9]. Exosomes regulate intercellular signaling and communication by delivering nucleic acids, proteins, and lipids to the recipient cell [10]. Exosomes are ubiquitous in body fluids such as urine, amniotic fluid, blood, and saliva. Their ability to function as modulator of target cells makes it crucial for maintaining homeostasis in multicellular organisms [11,12]. Exosomes play a critical role in intercellular communication, maintaining homeostasis, and regeneration of the nervous system [13]. Axonal regrowth involves a highly intricate process involving the activation of Schwann Cell (SC) and regulation of inflammatory processes [14]. Recent research reveals exosomes participating in these regenerative processes while exerting neuroprotective effects [15]. Exosomes are under investigation as a promising therapy for treating peripheral nerve injury. In this review, we provide an overview of the current research about the function of exosomes as well as their emerging role in peripheral nerve repair.

### Exosomes

**Figure 1:**
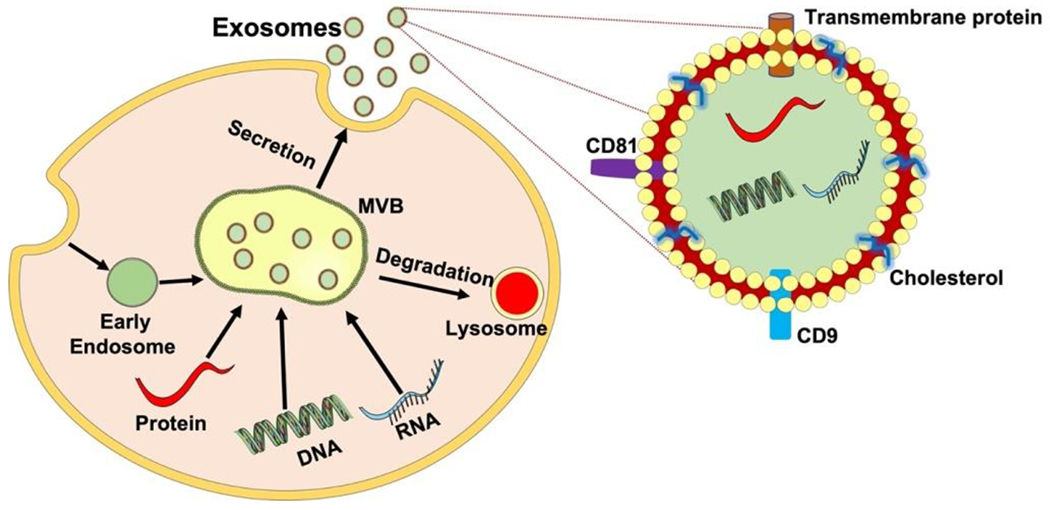
The process and structure of exosome secretion. The inward budding of the plasma membrane forms an early endosome. As the endosome matures, intraluminal vesicles are formed, and subsequent invagination leads to the formation of multivesicular bodies (MVBs). MVBs fuse with lysosomes and plasma membranes to release intraluminal vesicles into the surrounding environment as exosomes facilitating cell-cell and cell-extracellular matrix communication via its cargo delivery of protein, lipids, mRNA, and miRNA enclosed within their bilipid layer and induce therapeutic effects in peripheral nerve regeneration.

Extracellular Vesicles (EVs) are secreted by many cell types including MSCs, epithelial cells, immunocytes, and SCs [16]. EVs are membrane contained vesicles that can be divided into microvesicles (MVs) and exosomes [17]. Exosomes are created by inward budding inside endosomes, which result in the formation of multivesicular bodies (MVBs) that are able to fuse with the plasma membrane and released outside the vesicle, while MVs are budding vesicles that shed directly from the plasma membrane [18]. Additionally, exosomes can be differentiated from others by size. Exosomes are nanosized membrane vesicles that range from 40-150 nm in diameter, whereas MVs are roughly 100 to 1000 nm. The electron microscopic studies revealed the exosome morphology, which is described as “cup-shaped” [19]. Exosomes are endocytic vesicles derived from the membrane with a lipid structure consisting of phospholipids, cholesterol, ceramide, and saturated fatty-acyl chains [20,21]. The bilayer membrane enables protection and provides a controlled microenvironment allowing cargo to be moved across vast distances [22]. Secretion of these exosomes involves an intricate endocytic pathway. This begins with invagination from the plasma membrane into the cytoplasm. As the endosome buds inward to form intraluminal vesicles, they mature. At this stage in development, endosomes are called multivesicular bodies (MVBs). The MVBs then fuse with the cell membrane and released into the extracellular space. Some MVBs are transported to lysosomes where they are degraded [23]. The underlying mechanism of the biogenesis of MVB and intraluminal vesicle involves two unique molecular processes, namely the Endosomal Sorting Complex Needed for Transport (ESCRT) and the ESCRT-independent pathway [24]. As exosomes are released into the extracellular environment, they can interact with lipid ligand receptors and are subsequently internalized through fusion within the cell membrane (Figure 1) [23].

Furthermore, exosomes are abundant in a variety of specific proteins such as annexins, flotillin, and nucleotide guanosine triphosphatases (GTPases) that all play a critical role in membrane fusion and transport. Heat shock proteins (Hsp90 and Hsp70) also modulate intracellular trafficking. Tetraspanins (CD82, CD81, CD63, CD9) can regulate cell migration and signaling [25,26]. Exosomes also contain messenger RNA (mRNA), noncoding RNA (ncRNA), and micro-RNA (miRNA). These RNAs can be used and translated into recipient cells and can target genes in other cells [27]. Exosomes are emerging as a prominent form of cellular communication within the nervous system, mediating nerve remodeling, nerve protection, and synaptic plasticity [28]. Exosomes also have the potential as a therapeutic delivery system as they are able to pass the blood-brain barrier and deliver cargo to specific target cells [29]. Growing evidence also suggest the role of exosomes in promoting nerve regeneration in the peripheral nervous system [30]. The engineered exosomes, that are called “smart exosomes” by modifying the exosome-secreting cells or directly modifying isolated and/or purified vesicles, could effectively be used in the therapy following nerve injury [31]. This makes exosomal therapy a pivotal topic in the treatment of peripheral nerve injury.

### Exosomes and Schwann Cells

Schwann cells (SCs) are the glial cells of the peripheral nervous system [32]. They provide structural and mechanical integrity through establishing myelin sheaths around axons while increasing signal transmission velocity [33]. Following peripheral nerve injury, SCs dedifferentiate into progenitor type cells that involve in repair and undergo a phenotypic transformation [34]. These cells along with macrophages terminate myelin debris and create a microenvironment that allows axon repair to take place [35]. Additionally, cytokines, neurotrophins, and growth factors are synthesized from SCs to increase the survival of neurons [36]. As newly created axons come into contact, SCs undergo redifferentiation and accomplish nerve repair [37]. Recent research reveals exosomes can promote peripheral nerve repair through upregulating SCs. One study showed adipose MSC-derived exosomes are able to enhance the myelination, migration, and proliferation of SCs after they were internalized by SCs. This was accomplished by increasing the expression of the corresponding genes in vitro. A similar *in vivo* experiment revealed that the group treated with exosomes optimized functionality of SCs and led to increased rates of remyelination, axonal regrowth, and muscle restoration as opposed to the control group [38]. Additionally, it has been studied that adipose MSC-derived exosomes can decrease the rate of autophagy of SCs through downregulating karyopherin subunit α2 (Kpna2) expression via miRNA-26b. This ultimately has shown to improve the repair process of peripheral nerve injury [39]. MSC-derived exosomes can also improve proliferation of SCs through upregulation of anti-apoptotic Bcl-2 mRNA levels while downregulating the pro-apoptotic Bax mRNA levels. This subsequently provides a neuroprotective function following peripheral nerve injury [40]. Furthermore, adipose MSC-derived EVs has a promotional effect on SCs and can improve SC proliferation in a dose-dependent manner. This was done via adipose MSC-derived EVs entering SCs through endocytosis as opposed to binding or fusing with the plasma membrane of SCs. The miRNAs contained within the EVs impact gene expression in SCs in response to nerve damage [41]. Additionally, adipose MSC-derived exosomes were also noted to demonstrate SC proliferation and remyelination in 10 mm nerve defects in peripheral nerve injury murine models [42]. Collectively, these studies demonstrate exosomes exerting a neuroprotective effect by influencing SCs and can be a potential therapy for peripheral nerve injury.

### Exosomes and Axonal Regeneration

Axonal regrowth has been a subject of study as it is essential for functional recovery in damaged nerves. Axons must extend across damaged nerve sites and reconnect with distal nerves. Growth cones are distal tip expansions of regenerated axons that are able to guide and sense growth [43]. Filopodia are membranous protrusions that are extended by growth cones and lamellipodia interact with the surrounding environment [44]. The growth cone is able to sense the surrounding milieu through specialized structures like actin, neurofilament cytoskeleton proteins, and microtubules, all of which mediate axonal growth [45]. During peripheral nerve repair, adhesion molecules are expressed and can influence SC migration [46]. During earlier stages of this process, axons act as a guide for SC migration in the proximal nerves [47]. Axons are critical for SC repair and studies reveal when SCs lack axonal contact for long periods of time, their regenerative capacity diminishes [48]. Exosomes derived from SCs can modulate the regeneration process of axons. It has been established that exosomes from dedifferentiated SCs were internalized by axons which facilitated the survival of dorsal root ganglion neurons in vitro. Furthermore, regeneration of sciatic nerves has been researched and their ability to regenerate was further enhanced using SC derived exosomes. These exosomes contributed to a pro-regenerating phenotype of growth cones that inhibited the function of GTPase RhoA, a protein involved in growth cone collapse [49, 50]. It was also shown that the shift of SCs into a repair phenotype was accomplished by modifying the miRNA cargo of exosomes. Increased levels of miRNA-21 exhibited regenerative characteristics and are a key factor in repair SC exosomes. This regenerative capability was accomplished by downregulating PTEN and PI3-kinase activation in the nervous system [51]. Other than SC-derived exosomes, exosomes from MSCs have also shown promising data to regenerate nerves. For example, adipose MSC-derived exosomes increased rates of neuron outgrowth of dorsal root ganglion cells in vitro and enhanced repair of sciatic nerve injury [52]. Moreover, it has been studied that bone marrow MSC-derived exosomes increased axonal length and neurite growth through miRNA-mediated regulation of repair genes [53]. With these studies and their different results, it becomes imperative to explore the appropriate dosage of exosomes to achieve optimal repair in nerves. Another study revealed that fibroblast-derived exosomes modulated neurite growth and elongation of murine retinal ganglion cells by promoting Wnt10b and activating mTOR [54]. Gingival MSC-derived exosomes have also shown to increase dorsal root ganglion axon outgrowth [55]. Overall, these studies demonstrate the crucial role exosomes play in axonal regrowth and regenerative signaling.

### Exosomes and Regulating Inflammation

Nerve repair and regeneration involves a pathologic process of inflammation which influences the prognosis of peripheral nerve injury [56]. SCs secrete a variety of chemokines and inflammatory cytokines during Wallerian degeneration. Macrophages are then recruited to enhance the clearance of myelin debris while initiating an inflammatory cascade [57]. Macrophages play an imperative role in coordinating inflammation and the events involved for successful nerve regeneration [58]. Macrophages possess heterogenous phenotypes which include M1 activating cells and alternatively-activated M2 cells [59]. The M1 phenotype is able to secrete IL-1β, IL-6, TNF-α, and IFN-γ which results in further nerve damage. The M2 phenotype, however, is involved in the inhibition of the inflammatory response by secreting cytokines like IL-10 and IL-4 [60]. Their release of various growth factors also allows M2 macrophages to have a neuroprotective effect [61, 62]**.** During the earlier stages of inflammation, macrophages initially present as the M1 phenotype and later transform into M2 which decreases inflammation [63]. The inflammatory cascade aids in peripheral nerve repair, however, excess inflammation can lead to insufficient regeneration [64]. With an appropriate regulation of the inflammation that follows peripheral nerve injury, neuron apoptosis and axon demyelination can decrease [65]. Therefore, targeting the inflammatory response for peripheral nerve injury has become a therapeutic intervention that has been studied. Exosomes can modulate the immune reaction to peripheral nerve injury [66]. For example, MSC-derived exosomes have been shown to harness similar anti-inflammatory effects as parent cells, which provides a favorable environment for nerve regeneration [67]. One study demonstrated umbilical cord MSC-derived exosomes to promote healing of spinal cord injury through modulating the inflammatory response in the region of damage. Their findings illustrated that exosomes can influence macrophage polarization from M1 phenotype to M2 phenotype [68]. It has been shown that exosome therapy can also improve recovery by down regulating inflammatory cytokines like IFN-γ, IL-6, and TNF-α. Furthermore, bone marrow MSC-derived exosomes demonstrated neuroprotective effects by decreasing inflammation in murine models with traumatic brain injury through influence macrophage polarization [69]. Additionally, these exosomes have also been shown to modulate neurovascular remodeling and increased recovery in the diabetic murine model with peripheral neuropathy [70]. In a study using a sciatic nerve injury murine model, umbilical cord MSC-derived EVs were researched on peripheral nerve regeneration. This study concluded that EVs can decrease levels of IL-6, IL-1β, and increase anti-inflammatory cytokines such as IL-10 at distal nerve stumps [71]. SC-derived exosomes have also shown promising data and played a crucial role in modulating the inflammatory phase of nerve regeneration through the presence of α-Crystallin B and galectin-1 [72]. Overall, research on exosomes in peripheral nerve repair has demonstrated that control of neuroinflammation can lead to increased recovery in peripheral nerve injury.

### Exosomes and Vascular Regeneration in Peripheral Nerve Repair

The vascular network is imperative for nerve regeneration as it facilitates axonal growth during nerve repair [73]. Maintaining vascular integrity becomes integral following peripheral nerve injury and is another major target for therapy. MSC-derived exosomes have gained attention as paracrine promoters for angiogenesis and have been studied as a possible therapy option for peripheral nerve repair. Exosomes from pluripotent stem cell-derived mesenchymal stem cells influence angiogenesis by initiating the PI3K/AKT pathway in cells of endothelial origin [74]. Research has shown that miRNAs that are proangiogenic can be transported within endothelial cells through exosomes generated from MSCs and subsequently can improve vascularity post peripheral nerve injury [75]. Exosomes have also been shown to induce angiogenesis. A study involving intravenous infusion of MSC-derived exosomes exhibited improved neurite remodeling, angiogenesis, and overall recovery [76]. Similar results were shown in another study where MSC-derived exosomes promoted neurogenesis and angiogenesis in the murine models of peripheral nerve injury [77]. Moreover, bone marrow MSC generated exosomes have been shown to decrease ischemic brain injury by increasing revascularization of endothelial cells and decreased rates of neuron apoptosis through delivering miRNA-29b-3p. This might subsequently activate PTEN-mediated Akt pathway to carry out its protective and angiogenic promoting effects [78]. Endothelial progenitor cell generated exosomes have also reduced rates of neuron apoptosis and improved revascularization through the miR-126/PI3k pathway [79]. In summary, these studies portray exosomes derived from MSC origin as mediators of vascular endothelial cells which improve blood supply to peripheral nerves. Exosomes from MSC can provide new avenues of therapy for peripheral nerve injury repair through enhancing angiogenesis. However, further studies are needed to elucidate the full effects exosomes may have on angiogenesis in the setting of peripheral nerve injury.

### Challenges using Exosomes

Recent research revealed exosomes being a top contender for promoting peripheral nerve regeneration after peripheral nerve injury. Exosome therapy is expected to be a feasible therapeutic option; however, challenges remain that need to be addressed. The methods of obtaining and purifying exosomes are a major setback that hampers the application of exosomes in a clinical setting. Several kits for isolating exosomes have been created to combat this setback which have been proven to be effective and reliable [80]. Additionally, the heterogeneity of the cargo exosomes poses a challenge due to the variety of functions that various proteins and RNA molecules possess. Thus, regulation of exosomes to the desired target is a challenging process. Administration routes have also been a point of concern for exosome therapy. The effectiveness of administration in a clinical setting needs to be further studied. Research has shown nerve regeneration increased when exosomes were integrated into tissue engineered nerve grafts [81]. Exosomes also have the potential to replicate and propagate transmissible diseases and its mass production should not be ignored to ensure the safety of patients. Much work has yet to be done to overcome the various limitations in the therapeutic application of exosomes in a clinical setting.

## Conclusion

Exosomes have been widely studied as main modulators of tissue regeneration [82]. Exosomes exert their therapeutic effect by mediating intercellular communication. Their ability to transfer genetic material, proteins, and neurotrophic factors to axons allows restoration of homeostasis in the microenvironment of peripheral nerve injury. This allows axonal regrowth, hence promoting recovery after peripheral nerve injury. The regenerative effect of exosomes reinforces the paradigm that promoting nerve repair by MSCs is mediated by a paracrine pathway and provides new avenues for therapies [83]. The use of MSC exosomes can reduce the issues of stem cell transplant and in the future MSC exosome therapy represents a promising therapy for peripheral nerve injury repair. Although many studies show the use of MSC exosomes for peripheral nerve regeneration is effective, it would be best to reprogram or manipulate the secretome contents and functions by modifying the exosome-secreting cells or directly modifying the isolated and purified vesicles to develop the engineered exosomes for peripheral nerve regeneration. Obviously, further research is yet to be conducted to fully elucidate the potential of exosome therapy in a clinical setting.

## Data Availability

Not applicable since the information is gathered from published articles.
